# Evolution of Genome Size in Asexual Digital Organisms

**DOI:** 10.1038/srep25786

**Published:** 2016-05-16

**Authors:** Aditi Gupta, Thomas LaBar, Miriam Miyagi, Christoph Adami

**Affiliations:** 1 BEACON Center for the Study of Evolution in Action, Michigan State University, East Lansing, MI 48824 USA; 2 Department of Microbiology and Molecular Genetics, Michigan State University, East Lansing, MI 48824 USA; 3 Program in Ecology, Evolutionary Biology, and Behavior, Michigan State University, East Lansing, MI 48824 USA; 4 Department of Integrative Biology, University of Texas at Austin, Austin, TX 78712 USA; 5 Department of Physics and Astronomy, Michigan State University, East Lansing, MI 48824 USA; 6 Present Address: Present address: New Jersey Medical School, Rutgers University, Newark, NJ 07103, USA., ,

**Keywords:** Computational platforms and environments, Evolution

## Abstract

Genome sizes have evolved to vary widely, from 250 bases in viroids to 670 billion bases in some amoebas. This remarkable variation in genome size is the outcome of complex interactions between various evolutionary factors such as mutation rate and population size. While comparative genomics has uncovered how some of these evolutionary factors influence genome size, we still do not understand what drives genome size evolution. Specifically, it is not clear how the primordial mutational processes of base substitutions, insertions, and deletions influence genome size evolution in asexual organisms. Here, we use digital evolution to investigate genome size evolution by tracking genome edits and their fitness effects in real time. In agreement with empirical data, we find that mutation rate is inversely correlated with genome size in asexual populations. We show that at low point mutation rate, insertions are significantly more beneficial than deletions, driving genome expansion and the acquisition of phenotypic complexity. Conversely, the high mutational load experienced at high mutation rates inhibits genome growth, forcing the genomes to compress their genetic information. Our analyses suggest that the inverse relationship between mutation rate and genome size is a result of the tradeoff between evolving phenotypic innovation and limiting the mutational load.

**Supplementary information:**

The online version of this article (doi:10.1038/srep25786) contains supplementary material, which is available to authorized users.

## Introduction

Genome sizes evolve by various mechanisms, some of which are common to all domains of life (insertions and deletions) while others are seen in some taxonomic groups more than others (horizontal gene transfer in bacteria and transposable element activity in eukaryotes). While one might think that genome expansion leads to the acquisition of more protein-coding genes and functions, genome size does not strongly correlate with organismal complexity (the C-value paradox). Whole-genome sequencing data provide some explanation for this paradox: appreciable variation in eukaryotic genome sizes has been attributed to ploidy^1^, and to an expansion of non-coding DNA such as introns, intergenic regions, and repeats^2^. Yet, genome size also positively correlates with the number of protein-coding genes^2^, suggesting that larger genome size *is* a prerequisite for gaining new genes that could lead to phenotypic innovation.

The point mutation rate, relative frequencies of insertions and deletions (indels), and population size are three factors seen across the tree of life that are thought to influence genome size evolution. The negative correlation between genome size and point mutation rate is observed in all living organisms, from viruses to *Homo sapiens*^3^. However, a recent analysis based on more taxa found that this inverse relationship holds true only for prokaryotes and viruses, and that genome size and mutation rate are instead positively correlated in eukaryotes^4^. A high point mutation rate forces viruses to maintain small genome sizes in an effort to limit the number of deleterious mutations^5^. This selection pressure to reduce genome size is so strong that viruses eliminate non-functional sequences inserted into their genomes^6^ and lose an essential gene if it is transferred to the host genome^7^. This suggests that the point mutation rate and the evolution of genome size are inherently intertwined.

Population size, together with the point mutation rate and genome size, determines the mutation supply rate in an evolving population: if too many mutations are occurring, then reduction in any or all of point mutation rate, genome size, and population size can lower the mutation supply rate. Moreover, the effect of genetic drift is enhanced and purifying selection is weakened in small populations, allowing non-beneficial genome edits to persist for generations^8^. Lynch and Conery postulate that these—initially nonadaptive—edits can become a source of phenotypic innovation later on^2^ (this model, however, has been challenged^9^). In symbiotic bacteria, small population size and asexual reproduction cause bacterial genomes to shrink to the extent that they become 2–4 times smaller than the smallest genome seen in an independent-living organism^10^. In contrast, large population sizes in microbial populations weaken the effect of random drift, preventing accumulation of non-functional DNA and genome growth^11^.

In addition to point mutation rate and population size, biases in patterns of insertions and deletions (indel spectra) have been suspected to contribute to the variation in genome sizes we see today^12^. DNA loss via deletions is purported to be important in determining genome size, but this perspective is derived from analysis of a small number of eukaryotic genomes^13,14^. A strong deletion bias was found in 12 bacterial species as well^15^, the majority of which have transposable element (TE) activity. Thus, it is likely that deletions outnumber insertions in taxa where TE proliferation leads to significant increases in non-functional DNA. This explanation, however, does not apply to genome size evolution in early living organisms and in taxa where TE activity is absent, and it is not clear how primordial genome editing mechanisms shaped the diversity in genome sizes we see today.

Digital evolution provides an apt platform for understanding the evolutionary processes that determine genome size. While naturally evolving biological systems can take a very long time to show observable changes, the short generational time of digital organisms significantly reduces the time-scale of experiments to study evolutionary processes^16,17,18^. Avida is one such artificial life platform, where digital organisms are simple computer programs that compete for resources to replicate via a mutation-prone process, thus evolving under Darwinian dynamics^16,17,19,20^. The computer programs (or, ‘avidians’) contain a sequence of instructions that are executed to perform Boolean calculations (comparable to phenotypic traits in organisms) and to self-replicate by copying their instructions (with errors, similar to mutations in organisms) into a new avidian (see Methods for more details). As such, digital evolution is not a simulation of evolution, but rather an *instance* of evolution^21^ because avidians physically populate the computer’s memory, and reproduce mechanistically. We should think of avidians, therefore, as a “model organism” as opposed to a computational simulation^22^, capable of generating hypotheses that can be tested with biological organisms.

The ability to control the mutation rate, genome sizes (the number of instructions in an avidian’s genome), and population size allows an inquiry into the impact of mutation rate and indel spectra on the evolution of genome size. Avida has been previously used to test many evolutionary hypotheses that are difficult to test via biological experimental evolution, such as the evolution of genomic complexity^17,23^, the ‘survival of the flattest’ effect in genotypes evolving at high mutation rates^24^, adaptive radiation^25^, co-evolution as a driving force for higher phenotypic complexity and evolvability^26^, the time-dependent effect of genetic robustness on evolvability^27^, and how standing genetic variation and environment influence evolutionary response to environmental stimuli^28^ (among many others). Here we use Avida to investigate genome size evolution because in addition to tracking genome edits and their fitness effects, we can record the evolution of phenotypic traits and thus study the consequences of genome size evolution on phenotypic complexity.

Because avidians reproduce asexually and lack mechanisms of genome expansion such as TE activity, their evolutionary dynamics are most similar to those of viruses and prokaryotes. To examine the mechanisms of genome size evolution in asexual populations, we evolved populations of avidians within a range of mutation rates and followed the changes in their genome lengths, population fitness, genetic information, and phenotypic outcomes. Our results confirm that genome size is negatively correlated with mutation rate. By tracking the changes in genome size and the fitness effects of insertions and deletions that cause these changes, we find that insertions drive genome growth at low mutation rates, contributing to the evolution of phenotypic complexity via a two-step process: genome expansion followed by repurposing of the extra genome content to evolve new traits. Finally, we show that the mutational load at high mutation rates increases the selection pressure for reducing the genome size, resulting in smaller genomes with high information density. We conclude that genome size evolution is the result of a compromise between acquiring phenotypic complexity and restricting the mutational load.

## Results and Discussion

To test the role of mutational pressure in the evolution of asexual genomes, we evolved populations of avidians across a range of different point mutation rates (2.5 × 10^−3^ mutations per locus per generation to 0.1 mutations per locus per generation). With an ancestral genome size of 20 instructions, these populations had ancestral genomic mutation rates from 5 × 10^−2^ to 2.0 mutations per generation. These populations had a constant population size of 3600 individuals and evolved for 2 × 10^5^ generations. Selection coefficients of mutations are not pre-determined and are calculated as the effect of the mutation relative to the fitness of the ancestral organism. At the end of the experiment, a variety of traits from the genotype with the highest fitness were measured and used in the statistics below. For analyses involving insertions and deletions, the genotype’s evolutionary history was examined (see Methods for further details).

### Mutation rate is negatively correlated with genome size

**Figure 1:**
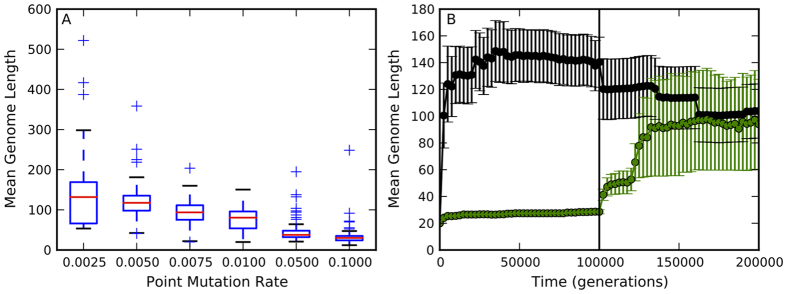
Point mutation rate is a strong determinant of genome size. (**A**) Genome size and mutation rate are negatively correlated in asexual populations. The initial conditions, i.e. the ancestral genome and population size, were identical for all point mutation rates in our study (0.0025, 0.005, 0.0075, 0.01, 0.05, and 0.1). The avidian populations at the lowest mutation rate (0.0025) are still evolving (mean population fitness is still increasing, Supplementary Fig. S1) after 200,000 generations, explaining the higher variation in genome length for this mutation rate. Red lines are median values from 100 replicates, while the upper and lower bounds of the box are the third and first quartile, respectively. Whiskers are either 1.5 times the the quartile value or the extreme value in the data, whichever is closer to the median. Plus signs are outliers. (**B**) The direct link between point mutation rate and genome size is further reinforced by switching the point mutation rate of population evolving at 0.0025 to 0.1 after 100,000 generations (black circles), and vice versa (green circles). The black line represents the generation where the mutation rates were switched. The long genomes shrink when mutation rate is increased and short genomes expand when mutation rate is decreased. Error bars represent ±1 SE. Values represent the mean genome length across the population, averaged over 20 replicates.

We found that genome size is negatively correlated with the mutation rate (Fig. 1A; Spearman’s *ρ* = −0.72, *p* < 3.6 × 10^−97^). The mean population fitness also increased as the avidians’ genomes grew (Supplementary Fig. S1). The evolved genomic mutation rates ranged from 0.13 to 24.85 (the genomic mutation rate was <2 for the lowest four point mutation rates). These genomic mutation rates are comparable to those seen in RNA viruses (0.025 in Influenza B virus, 1.1 in Hepatitis C Virus, and 4.6 in Bacteriophage Q*β*)^29^. Avidians did not evolve a constant genomic mutation rate in our experiments, as Drake observed in DNA microbes and RNA viruses^30,31^ and Knibbe *et al.* reported in their digital evolution experiments^32^. A constant rate of genomic mutation is also not observed across the tree of life^3^.

To test how genome size responds to changes in mutation rate, we performed experiments where we switched the mutation rates of the avidians evolving at the lowest (0.0025) and the highest (0.1) point mutation rate after 100,000 generations. We found that the longer genomes that initially evolved at the low mutation rate began to shrink and those evolved at the high mutation rate began to expand (Fig. 1B), further establishing the direct influence of mutation rate on genome size.

Since the ancestral genomes and population size were identical in all experiments, this negative correlation is independent of the effect of population size and the initial genomic content. By fixing the population size, we separated the influence of population size from that of mutation rate on genome size evolution, since it has been shown that population size influences genome size evolution as well^2^.

### Large genomes carry more genetic information

While genome expansion does not necessarily increase the number of functional sites in the genome, complex organisms are likely to have a higher amount of genetic information encoded into their genomes, which requires larger genomes. For example, even though *C. elegans* has a similar number of genes to *H. sapiens* (19,957 genes in the nematode compared to 20,181 in humans), the nematode has 20% less intergenic DNA and their mean intron size is 1/20th of that of humans^1^. On the premise that humans are more complex than *C. elegans*, one can argue that the expansion of non-coding DNA is at least partly responsible for this significant increase in complexity. Large proportions of the human genome are transcribed^33^, although much of this transcription is likely to be spurious and non-functional^34,35^. However, some of this transcription likely contributes to the non-coding RNA pool of the cell that regulates expression of protein-coding genes and participates in other cellular processes^36^. Introns are not always junk-DNA and contribute to the evolution of complexity in eukaryotes^37,38^. About 20% of the pseudogenes are transcribed in humans^39^, and are differentially expressed in cancers and viral infections^40,41^, indicating that some of these pseudogenes may be functional. Thus, genome expansion, even if primarily consisting of non-coding DNA, likely increases the *potential* for future increases in the number of functional sites in the genome. Even if some of this inserted DNA is non-functional at the outset, evolution can repurpose it to achieve higher organismal complexity and genetic information^42,43^.

**Figure 2:**
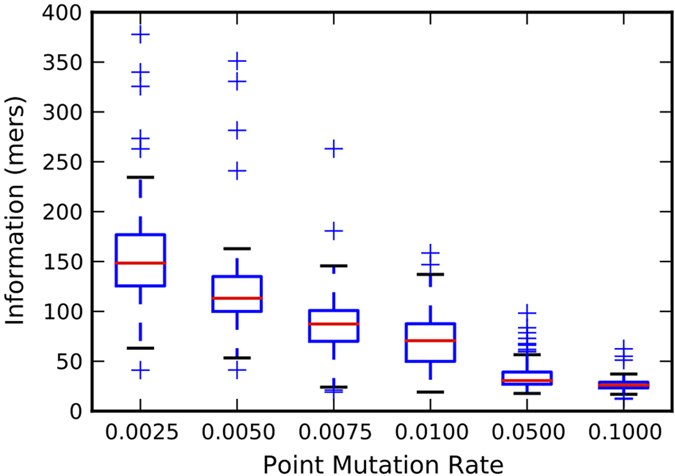
Genomes evolved at low mutation rates had higher genetic information (number of essential sites in the genome, see Methods) than genomes evolved at high mutation rates. The information measure is reported for the fittest genotype in each of the 100 replicate populations. Red lines are median values from 100 replicates, while the upper and lower bounds of the box are the third and first quartile, respectively. Whiskers are either 1.5 times the the quartile value or the extreme value in the data, whichever is closer to the median. Plus signs are outliers.

In our experiments, avidians that evolved long genomes at low mutation rates had higher genetic information content (number of essential sites in the genome) than those that evolved at high mutation rates and had shorter genomes (Fig. 2; Spearman’s *ρ* = −0.86, *p* < 6.4 × 10^−180^). The longer genomes also evolved more traits, which are the computational equivalent of biological pathways that lead to observable phenotypes (see Methods for an explanation of traits, and Supplementary Fig. S2). The mean population fitness was also inversely related to mutation rate, although the mean fitness of populations evolving at a point mutation rate of 0.0025 was still increasing after 200,000 generations (Supplementary Fig. S1). This suggests that larger genome size is a necessary, if not sufficient, requirement for evolving phenotypic novelty. The avidians on average evolved fewer traits when the point mutation rate was switched half-way from 0.0025 to 0.1, and evolved more traits when mutation rate was switched from 0.1 to 0.0025, emphasizing the relationship between genome size, mutation rate, and phenotypic complexity (Fig. 1B and Supplementary Fig. S3).

### Beneficial insertions drive genome expansion at low mutation rates

**Figure 3:**
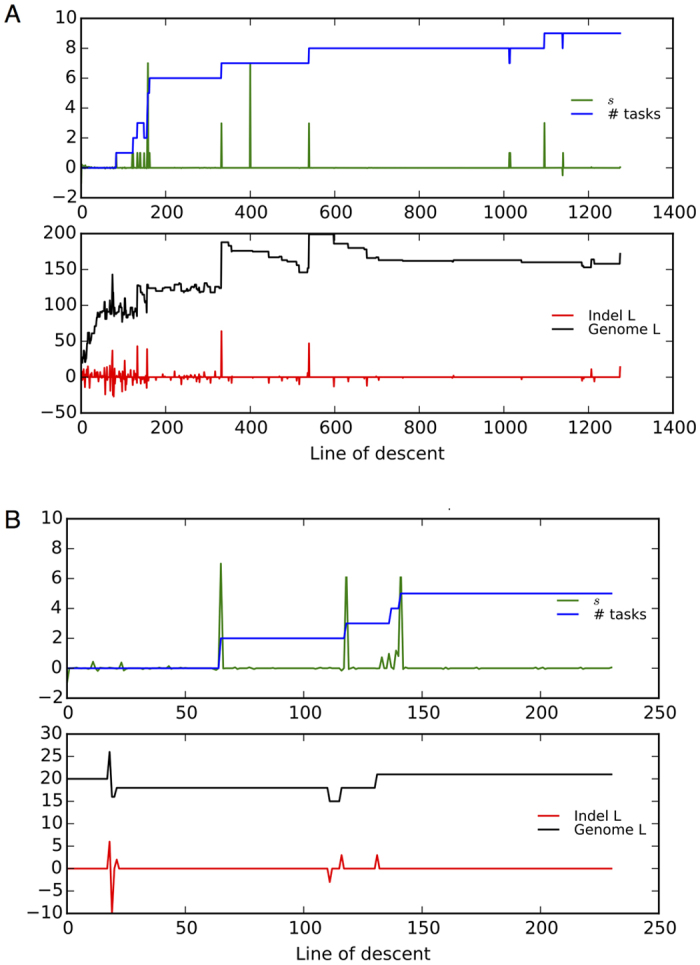
The line of descent (LOD) of the most fit genome is shown for a single replicate population evolving at the lowest (0.0025, **A**) and the highest (0.1, **B**) point mutation rate in our study. The fitness effects of genome edit events (insertions, deletions, base substitutions) are shown in green, the number of evolved traits is shown in blue, the size of indels is shown in red, and the genome length is shown in black. At low mutation rate (top panel, **A**), new traits (in blue) often evolved following beneficial genomic events (green spikes), and are sometimes concurrent with insertion events (red spikes). These beneficial insertions appear to increase the genome size (black line) over time. At the high mutation rate (bottom panel, **B**), insertion events are not as frequent as at low mutation rates (also see Supplementary Fig. S4), with genome size staying relatively constant. The line of descent (LOD) maps for other mutation rates can be found in Supplementary Fig. S5.

**Figure 4:**
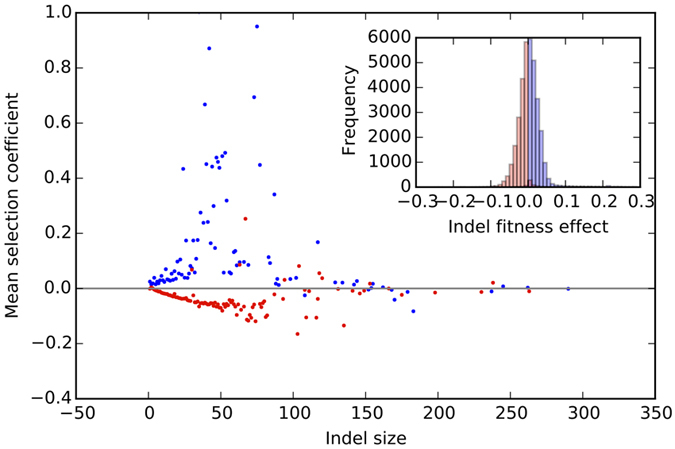
The average fitness effect of insertions (blue) and deletions (red) as a function of indel size is shown for 100 replicate populations evolving at the point mutation rate of 0.0025. Indels above the gray line (*s* = 0) are beneficial and those below the gray line are deleterious. Small insertions (blue dots) are usually beneficial, while small deletions (red dots) are usually deleterious. The inset plot shows the histograms of fitness effects of insertions (blue bars, total 19,262 insertions) and deletions (red bars, total 16,998 deletions) along the line of descent in 100 replicate populations. Insertions (blue bars) are usually beneficial (*i.e.*, fitness effect > 0), and deletions (red bars) are usually deleterious (fitness effect < 0). The two distributions are significantly different (Kolmogorov-Smirnov two-sided test, *p* < 1 × 10^−100^).

To understand how genomes gain meaningful increases in size, we followed the genome edits (indels and mutations; only the indel size is considered and not the sequence inserted/deleted or the context where the indel occured), the corresponding effect on fitness (*s*), the number of traits evolved, and overall genome size along the line of descent (LOD, see Methods) in avidians evolving at different mutation rates (Fig. 3). At the lowest point mutation rate in our experiments (Fig. 3A), the beneficial changes in the genome (green spikes) often align with evolution of new traits (blue line), as well as with insertions in the genome (red spikes). Insertions are largely beneficial compared to deletions at low mutation rate (Fig. 4). Phenotypic innovation (evolving a new trait) was preceded by insertion events 87% of the time (within the previous 20 ancestors along the line of descent), while deletions preceded innovation 60% of the time (null hypothesis: presence or absence of insertions is irrelevant to trait evolution, rejected with *p* < 1.0 × 10^−100^, *χ*^2^ test statistic = 2.23 × 10^5^). Thus, avidian genomes are likely to evolve new traits after an insertion event, suggesting that phenotypic innovation happens in a two-step process: genome expansion followed by evolution of a new trait by substitutions. Insertions are not deleterious per se (inset plots in Fig. 4) and thus persist on the line of descent. In fact, these inserted sequences may serve as substrates for evolving new phenotypic traits later on, contributing to an increase in fitness and phenotypic complexity. In contrast, indels are infrequent at high mutation rates on the line of descent (Fig. 3B, also see Supplementary Fig. S4). As a result, the genomes do not grow and evolve fewer traits compared to the genomes evolved at low mutation rates.

This prominent role of beneficial insertions in the genome evolution of asexual organisms is in contrast to how genome sizes are shaped by DNA loss in eukaryotes. The reported biases in indel spectra (rarity of long insertions and abundance of short deletions) are seen primarily in eukaryotic genomes^44^. Yet, a thermodynamic argument suggests that large indels are likely to increase genome size, since insertion events require only one breakpoint in the genome rendering large insertions less disruptive than large deletions^14,44^. By the same argument, DNA loss is more likely to happen by small deletions to minimize the fitness cost to the organism. Thus, while eukaryotic genomes may evolve by rapid expansion due to whole genome duplication events and TE proliferation, asexual populations such as RNA viruses may have grown their genomes gradually via beneficial insertions. However, gradual increases in avidian genomes at low mutation rates is still followed by small deletions that fine-tune the genome size (Fig. 3).

### High mutation rates force genomes to be small and informationally dense

**Figure 5:**
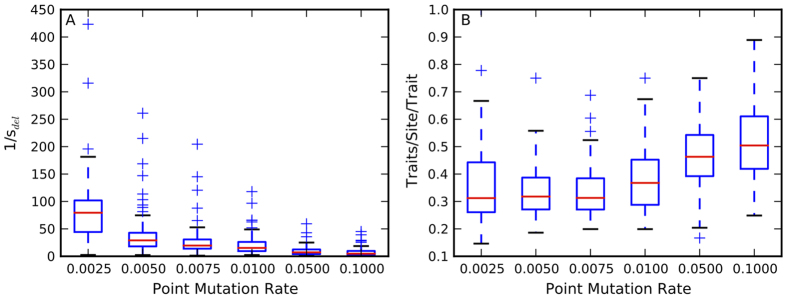
Deleterious mutations at high mutation rates are more costly due to informationally dense genomes. The inverse of the harmonic mean of deleterious selection coefficients for the fittest genotype from each replicate shows that deleterious mutations are costlier at high mutation rates (**A**). This can be explained by the high coding density in these genomes (**B**). Traits/site/trait represents how many traits are encoded per site, normalized by the total number of evolved traits, and thus is a measure of coding density of the genome. Red lines are median values from 100 replicates, while the upper and lower bounds of the box are the third and first quartile, respectively. Whiskers are either 1.5 times the the quartile value or the extreme value in the data, whichever is closer to the median. Plus signs are outliers.

If beneficial insertions drive genome expansion at low mutation rates, what keeps genomes small at high mutation rates? We find that the fitness cost of deleterious mutations is high at high mutation rates (Fig. 5A; Spearman’s *ρ* = −0.71, *p* < 1.1 × 10^−90^). Since genotypes evolving at high mutation rates are compact, genetic information is forced to be distributed over a small number of sites (Fig. 5B), as in the overlapping genes commonly seen in viral genomes. A deleterious mutation at a single such site can unfavorably affect multiple traits, increasing the overall fitness cost of deleterious mutations. Digital evolution experiments also find that gene knockouts are more deleterious when pleiotropy is high, as is common in compact genomes^45^. Thus, not only is the mutational load high at high mutation rates, the deleterious mutations are costlier than they are at low mutation rates. This compounding factor only strengthens the selection pressure to decrease mutational load by reducing genome size, especially since population size is fixed in our experiments.

It should be noted that mutation rate can itself evolve if an increased mutation rate facilitates adaptation (reviewed in^3,46^). For example, a mutator strain of *E. coli* with a higher mutation rate than the wild-type bacteria showed the ability to adapt faster^47^. Even though the majority of mutations are deleterious, the ability to quickly find adaptive beneficial mutations was enough to increase the population of the mutator strain relative to the wild-type^47^. However, this evolutionary advantage is short-lived and disappears once the beneficial mutations are found and there is no more fitness peak to climb^47,48^. The mutator strain also does not propagate faster than the wild-type when a higher mutation supply is achieved by increasing the population size^47,48^. Furthermore, environmental stresses such as starvation can trigger a response in bacteria that elevates the mutation rate, making it possible to quickly find beneficial mutations to adapt to temporarily adverse conditions^49^.

Since a high mutation rate increases the mutational load in an evolving population, it makes sense that when the environmental stress is no longer present, the mutation rate would revert to the lower level. After all, the fitness cost of accumulating deleterious mutations would be too high if the rapid rate of adaptation afforded by high mutation rate is not needed. Mutator strains in well-adapted bacterial populations evolve decreased mutation rates as the opportunity for adaptation diminishes^50^, an observation supported by digital evolution experiments^51^. Perhaps a continual need for adaptation is responsible for consistently high mutation rates in viruses, parasites, and sometimes in pathogenic bacteria where rapid adaptation to host immune responses is critical for surviving such an evolutionary arms race^3,52,53,54^. The selection pressure to adapt quickly to a changing environment appears to trump the selection pressure to decrease mutational load by minimizing the mutation rate. However, mutational load can restrict virus adaptability due to an abundance of deleterious mutations^55^. Thus, the compromise between evolutionary forces for reducing the mutation load and maintaining high adaptability might shape the genome size and information density in RNA viruses.

## Conclusions

While empirical studies reveal significant aspects of genome size evolution, digital evolution systems provide an opportunity to observe evolution-in-action and to manipulate evolutionary parameters in ways that allows exploring the relative importance of the many evolutionary forces that simultaneously act on genomes. Comparative genomics analyses have unearthed important relationships between population size, mutation rate, gene content, genome size, and their combined influence on evolution of complexity. Digital evolution experiments complement these retrospective observations by investigating evolutionary processes that are difficult to test experimentally.

In our experiments across a range of mutation rates, we find concurrence with the empirical finding that the point mutation rate is negatively correlated with genome size. By tracking the genomes along the line of descent, we find insertions to be significantly beneficial compared to deletions, suggesting that before the advent of complex mechanisms of genome edits such as TE activity, beneficial insertions drove genome expansion. That these insertions are followed by phenotypic innovations further explains why insertions are evolutionarily favored in asexual populations. At the same time, the point mutation rate influences genome size via the mutational load. Thus, unless a high mutation rate provides a critical evolutionary advantage such as rapid adaption to a temporary environmental stress, the selection pressure to reduce the mutational load forces genomes to shrink at high mutation rates. This shrinkage results in genomes packed with genetic information, and this compactness likely increases the fitness cost of deleterious mutations, further compounding the severity of the mutational load. Still, a high point mutation rate is frequently seen in natural populations, especially in viruses, suggesting that the selection pressure to maintain high evolvability (for example, against a highly adaptive host immune system^56^) can take precedence over the pressure to reduce mutational load in ensuring virus propagation.

It is natural to wonder whether evolution experiments with digital organisms can truly shed light on biological evolutionary processes. To some extent, this skepticism is no different from wondering whether evolution experiments with *E. coli* bacteria can shed light on the evolution of, say, elephants, as Monod famously quipped. Model organisms provide the opportunity to obtain results in well-characterized settings^57^, and invite us to interpret these results in a broader context. In this respect, we may find that “what is true for digital organisms may be true for *E. coli* only more so”^58^, or the experiments could offer up insights into why and how this model organism is fundamentally different from others. However, as an experimental system the outcome of experiments are reproducible and specific: it is not possible to obtain “any result one might want”, just as this is not possible when evolving bacterial or fungal model organisms.

In conclusion, our analyses of asexual populations evolving at fixed point mutation and indel rates reveal the fundamental roles that indel spectra and mutational load play in determining genome size and phenotypic diversity. The evolution of genome size is a complex phenomenon, especially in eukaryotes due to TE activity and expansion of non-coding DNA. Future investigations into eukaryotic genome size evolution by including recombination and TE activity in digital evolution platforms will allow comparisons with asexual genome size evolution, and can shed light on evolution of complex genome editing mechanisms.

## Methods

### Avida digital evolution platform

Avida is a digital evolution platform that provides an environment within which digital organisms, using sets of instructions analogous to codons, experience selective pressures to evolve genes that encode logical operations^16,19,59^. These digital organisms are simple computer programs (avidians) that compete for the resources needed to self-replicate via error-prone mechanisms. The avidian genome consists of computer instructions (thus, avidian genome length = number of instructions) that are executed during its life cycle to perform Boolean logic calculations, as well as to replicate its genome. Since evolution in Avida comprises genetic variation affecting the ability to evolve phenotypic traits and to replicate, differential fitness dependent on this heritable variation and competition for computational resources causes avidians to undergo evolution very much like biological populations.

The Avida world consists of a 60 × 60 toroidal grid with at most one avidian per cell, resulting in a fixed population size of 3600. Each child avidian is placed in any one of the 3600 cells after successful replication (although new offspring are preferentially placed in empty cells if available), making the population well-mixed. When the population is at its carrying capacity, the avidian occupying the cell chosen for a new offspring will be removed from the population. This random selection of individuals for removal adds an element of genetic drift to avidian populations.

Absolute time in Avida is divided into updates. During each update, the population executes 30 × *N* instructions, where *N* is the population size. The resource necessary to execute these instructions (comparable to energy units in cells–ATP) is called the “Single Instruction Processing Unit” (SIP), which are distributed across the population. How these SIPs are distributed to the individuals in the population is dependent on a characteristic possessed by each individual, called ‘merit’. Merit is increased when an organism is capable of performing logic calculations, a process that is the computational equivalent of metabolic processes in biochemical cells (see below).

In a clonal population, every individual will obtain on average 30 SIPs per update. However, if one individual has a greater merit than others in the population, it is expected to receive more SIPs per update than the other individuals. This allows it to execute and copy its genome faster than other individuals. Therefore, as reproduction speed is the primary target of selection in this type of simple environment, increased merit results in increased fitness, and organisms with an increased merit will be under positive selection. In our experiments, we record data every generation, starting from the ancestral population, which marks generation 0. All progeny of the ancestral population constitute generation 1, and so forth.

Avidians increase their merit through the evolution of phenotypic traits. These traits are the ability to perform Boolean logic computations. In the default Avida environment (the “Logic-9” environment^20^) populations can evolve up to 9 of these traits. Performing these traits result in a multiplicative increase in an individual’s merit (ranging from a multiple of 2 for simple traits to 32 for the most complex trait). The evolution of these traits require many point mutations and a genome size large enough to contain the instructions necessary to perform these computations. Because these traits increase merit, and thus replication speed, the evolution of these traits are also under strong selection. Each individual can perform each trait once during their lifespan, and there is no limit to the number of times a trait can be performed in a population. Because an individual’s performance of a trait does not limit the others in the population, there is only one niche in the environment. Therefore, fitness is frequency-independent.

During an avidian’s lifespan, it will eventually start to undergo genome replication. As it copies its genome’s instructions into a blank daughter genome, some instructions may be copied inaccurately at a point mutation rate set by the experimenter. Additionally, insertion and deletion mutations can occur either during genome replication or during genome division into new daughter genomes. In the experiments performed here, insertion and deletion mutations (indels) were enacted upon genome division. Genome sizes can change every generation by at most 10% (the default is a maximum change of 100%). For every indel, two spots in the genome were randomly selected. If the indel was a deletion, everything between those two spots was deleted. If the indel was an insertion, that section of the genome was duplicated. Insertions and deletions occurred at equal frequencies in our experiments.

### Experimental Design

To test the role of the mutation rate in driving genome size evolution, we evolved 100 replicate populations at various point mutation rates (*μ* = {0.0025, 0.005, 0.0075, 0.01, 0.05, 0.1}) for 200,000 generations. Insertions and deletions occurred with equal frequency at a constant rate of 0.05 indels per generation. Indel size was uniformly distributed, with genome size changing at most by 10% in any given generation. All populations were initialized with an identical ancestral genome of size 20. Population size was fixed at the default 3600 individuals. There was no structure in the evolving populations (i.e. a well-mixed environment). An additional 40 populations were evolved for 200,000 generations where the mutation rates were switched after 100,000 generations as follows: 20 populations that initially evolved at a point mutation rate of 0.0025 were switched to a point mutation rate of 0.1 after 100,000 generations, and the remaining 20 populations were switched from point mutation rate of 0.1 to 0.0025 after 100,000 generations.

### Line of Descent

To track the effect of genome edits on genome size and phenotypic evolution, we analyzed the Line of Descent (LOD) of the fittest individual from each replicate population at the end of the evolution experiments. A LOD is a lineage of every ancestor of the evolved genotype that had the highest fitness at the end of 200,000 generations. It tracks every genome edit (and its corresponding effect on fitness) that was fixed in the lineage. This genotypic “fossil record” allows identifying those mutations that lead to evolutionary innovations and determine the respective role of insertions and deletions in genome size evolution.

### Data Analysis

We calculated statistics at both the population level and for individual genotypes. The mean genome length and the mean fitness was calculated by averaging the relevant values across all genotypes in each population which was then averaged over 100 replicate populations. For the rest of our reported data, we calculated statistics from the fittest genotype in the final evolved population. A genotype’s information content was estimated as , where L is the genome size, 26 is the alphabet size for avidian genomes, and *ν*(*i*) is the number of mutations that are neutral or beneficial (see^17^ for further explanation of this estimation). Thus, information content is a measure of the number of essential sites in a genome. The number of phenotypic traits a genotype possesses is calculated as the number of different boolean logic calculations it can perform.

Selection coefficients of mutations were calculated as the ratio of the mutant’s fitness to its ancestor’s fitness minus 1 (i.e., the relative change in fitness). Fitness in Avida is estimated as a genotype’s merit divided by its replication time: the number of instruction executions, i.e., SIPs, needed to successfully copy its genome. In other words, in a population consisting of genotypes with the same merit, an avidian’s fitness is simply the number of offspring generated per unit time (that is, per update).

The “traits-per-site-per-trait” measure is determined by performing knockout mutations at every site in the genome and then counting the number of traits that are lost due to each knockout mutation (lethal knockouts are not considered). This gives the number of traits that use each genomic site, and the average of this quantity over the length of the genome gives the overall number of traits encoded per site. The normalized trait/site/trait is then calculated by dividing the traits/site by the total number of traits evolved by the genome.

## Additional Information

**How to cite this article**: Gupta, A. *et al.* Evolution of Genome Size in Asexual Digital Organisms. *Sci. Rep.* **6**, 25786; doi: 10.1038/srep25786 (2016).

## Supplementary information

Supplementary Information (PDF 536 kb)
