# Supplementary material for: Evolution of Genome Size in Asexual Digital Organisms
Source: Sci Rep. 2016 May 16;6:25786. doi: 10.1038/srep25786 (PMC4867773; doi:10.1038/srep25786)
Supplement: Supplementary file 1 — Supplementary Information (PDF 536 kb) [file 41598_2016_BFsrep25786_MOESM121_ESM.pdf]

# Evolution of Genome Size in Asexual Digital Organisms
# Supplementary Materials

Aditi Gupta*[1,2,§], Thomas LaBar[1,2], Miriam Miyagi[3], and Christoph Adami[1,2,4]

[1]BEACON Center for the Study of Evolution in Action, Michigan State University, East Lansing, MI 48824
[2]Department of Microbiology and Molecular Genetics, Michigan State University, East Lansing, MI 48824
[3]Department of Integrative Biology, the University of Texas at Austin, Austin, TX
[4]Department of Physics and Astronomy, Michigan State University, East Lansing, MI 48824
[§]Present Address: New Jersey Medical School, Rutgers University, Newark, NJ 07102.

*aditi9783@gmail.com

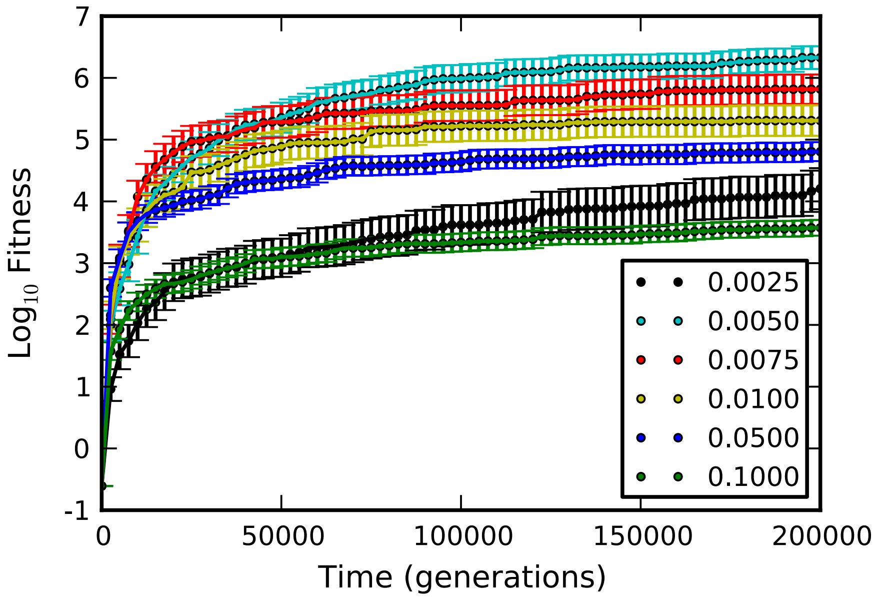


Figure S1: Increase in population fitness over 200,000 generations is shown for six point mutation rates (0.0025, 0.005, 0.0075, 0.01, 0.05, and 0.1). The $log_{10}$ of fitness is averaged over 100 replicate populations. Error bars represent $\pm$ 1 SE.

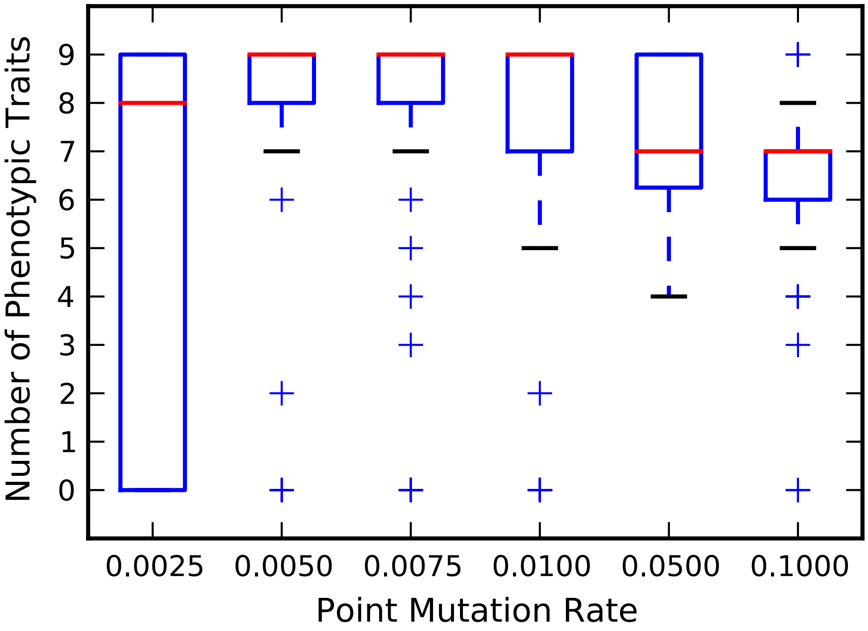


Figure S2: Number of traits evolved by avidians at six different point mutation rates (maximum number of traits that can be evolved is 9). Red lines are median values from 100 replicate populations, while the upper and lower bounds of the box are the third and first quartile, respectively. Whiskers are either 1.5 times the the quartile value or the extreme value in the data, whichever is closer to the median. Plus signs are outliers.

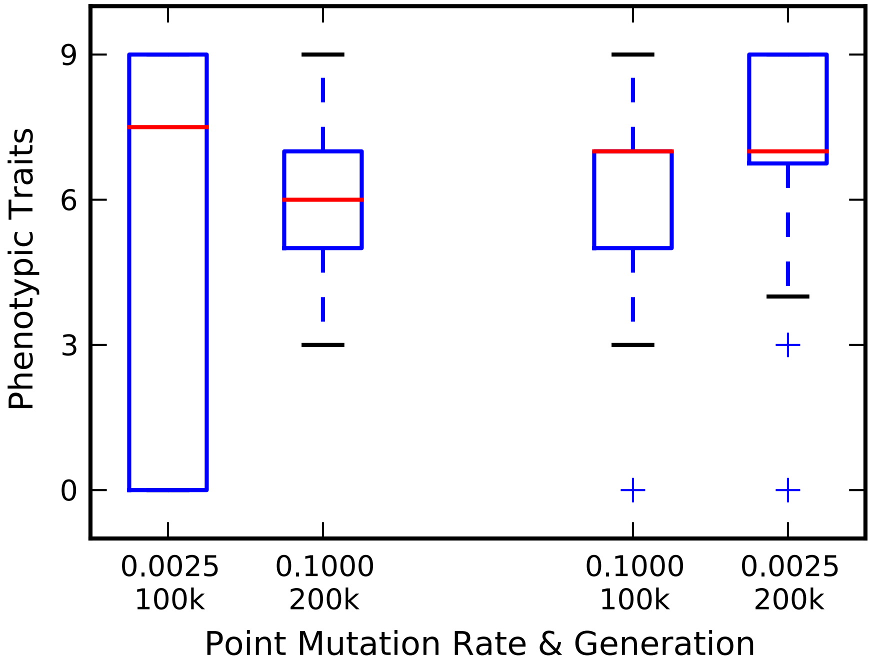


Figure S3: The number of traits evolved by avidians after mutation rate was switched at the mid-point (at 100,000 generations) in the 200,000 generation long simulations. Left side of the figure shows the statistics for number of traits evolved by the population evolving at point mutation rate of 0.0025, and the number of traits the same population evolved after evolving at mutation rate of 0.1 for 100k generations. Right side shows the reverse scenario: traits evolved by population evolving at point mutation rate of 0.1 at 100k generations, and after mutation rate is switched to 0.0025 for additional 100k generations. Red lines are median values from 20 replicate populations, while the upper and lower bounds of the box are the third and first quartile, respectively. Whiskers are either 1.5 times the the quartile value or the extreme value in the data, whichever is closer to the median. Plus signs are outliers.

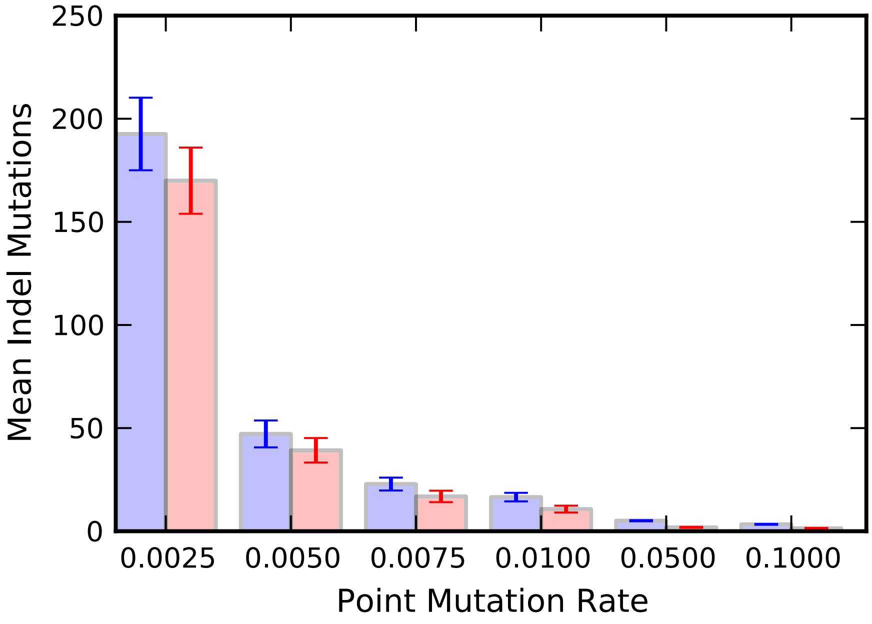


Figure S4: Insertions (blue bars) are more common than deletions (red bars) at all mutation rates, and frequency of indels decreases as mutation rate increases. Average values over 100 replicates is reported with error bars showing ± 1 SE.

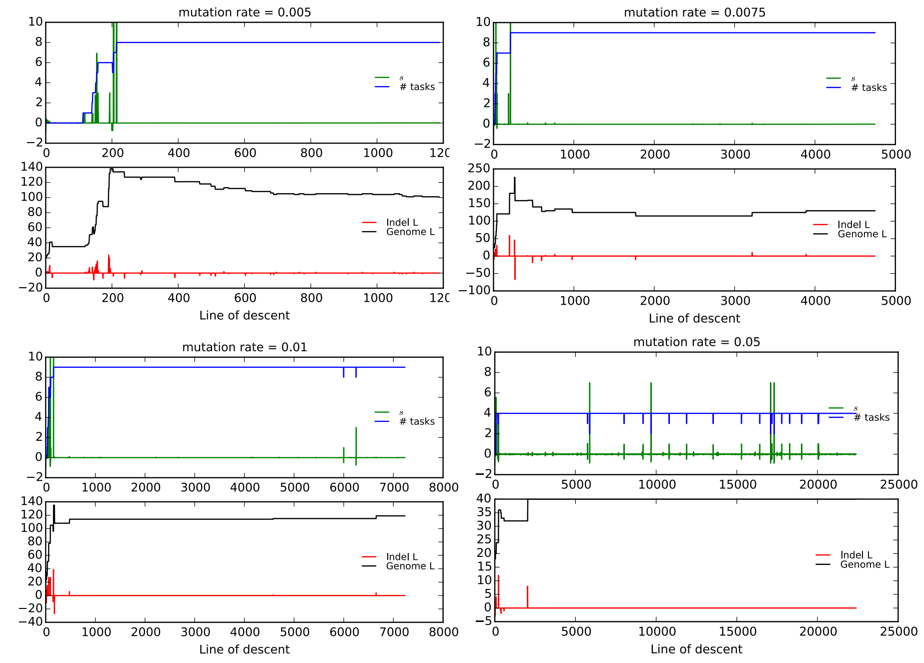


Figure S5: The line of descent (LOD) of the most fit genome is shown for a single replicate population evolving at the point mutation rates 0.005, 0.0075, 0.01, and 0.05. The fitness effects of genome edit events (insertions, deletions, base substitution) is shown in green, the number of traits evolved over time is shown in blue, the size of indels is shown in red, and the genome length is shown in black.
